# Chiropractic evaluation in newborn foals: A preliminary study

**DOI:** 10.1016/j.vas.2025.100495

**Published:** 2025-08-15

**Authors:** Ilaria Arena, Silvia Signor, Jole Mariella, Aliai Lanci, Francesca Freccero, Castagnetti Castagnetti

**Affiliations:** a Private practitioner, Ravenna, Italy; b Private practitioner, Bologna, Italy; c Department of Veterinary Medical Sciences, Bologna Università, Via Tolara di Sopra 50, Ozzano dell’Emilia 40064, Italy; d Health Sciences and Technologies Interdepartmental Center for Industrial Research (CIRI-SDV), Bologna University, 40100, Italy

**Keywords:** Foal, Chiropractic, Motion palpation, Perinatology

## Abstract

This paper describes the more frequent chiropractic alterations in healthy and sick foals. The assessment is performed through a motion palpation exam, which locates the hypomobile joints along the skeleton. The motion palpation exam allowed the identification of multiple hypomobile areas in neonatal foals. The sick foals presented a statistically significant higher number of hypomobile joints in the cervical, thoracic, lumbar regions and limbs compared to the healthy foals. The most frequent alterations in sick foals involved the atlantooccipital joint, C5-C6, T14-T15, L5-L6, the sacrococcygeal joint and the scapulohumeral joint. In both groups, high prevalence of alterations occurred at the cervical and sacroiliac joints. The results show that some regions can be highly stressed during the perinatal period, especially in sick foals. Possible explanations are discussed. Chiropractic evaluation and subsequent manipulation could be routinely used to improve neonatal health, especially in sick foals.

## Highlights

• Chiropratic assessment is performed through a motion palpation exam.
• Sick foals presented a higher number of hypomobile joints.
• In sick and healthy foals alterations of cervical and sacroiliac joints occurred.
• Some regions can be highly stressed during the perinatal period.
• Chiropractic manipulation could be applied to restore the physiological motion.

## 1 Introduction

In the last decades, the interest in an integrated veterinary approach has been increasing. Veterinarians have been discovering new complementary therapeutic tools.

Chiropractic is a manipulative technique based on a very specific, short lever, high velocity controlled forceful thrust taking the joint to its paraphysiologic space. This is the space laying beyond the passive range of motion, between the elastic barrier of soft tissues and the anatomic limit of osseous segments (Sandoz, 1976; Haussler, 1999; Haldeman, 2000; Alcantara et al., 2003; Cleveland, 2003). The thrust, called “adjustment”, is applied on a motion unit (a functional unit made up of two adjacent articulating surfaces and the connecting tissues binding them) expressing a limited range of motion. The range of motion is evaluated by the chiropractic assessment or “motion palpation exam”. The adjustment aims to restore the normal range and pattern of motion, joint mechanics, load distribution and joint neurology (Cleveland, 2003). In fact, chiropractic theories consider articular dysfunction as an alteration of normal neurological patterns (Haussler, 1999). The chiropractic manipulation re-establishes the physiological movement optimizing the body biomechanics and the function of the nervous system to promote and preserve health (Alcantara et al., 2003).

Many human studies have reported a therapeutic effect of chiropractic in several medical conditions like back pain and fibromyalgia, and it is widely used in neonatology to manage gastrointestinal pain, constipation and breast-feeding issues (Colloca & Keller, 2001; Borowitz et al., 2003; Alcantara & Mayer, 2008; Browning & Miller, 2008; Alcantara et al., 2015). A recent meta-analysis provided an overview of the evidence regarding the effectiveness of chiropractic, osteopathic and spinal manual therapy in infants, children and adolescents (Driehuis et al., 2019). According to this analysis, the treatment protocols showed a moderate to poor usefulness. The complexity of the different human pediatric pathologies and the lack of information encourage controlled studies focusing on the effectiveness and safety of these techniques (Driehuis et al., 2019).

In equine sport medicine, chiropractic is becoming popular, and it is mainly applied to treat back pain conditions (Saunders et al., 2005; Alvarez et al., 2008; Haussler, 2010, 2016, 2018). There are no studies about chiropractic applications in equine neonatology. The neonatal period is crucial for the foal’s musculoskeletal structure which is subjected to various changes and stimuli.

This study aims to describe the primary chiropractic alterations affecting the spine and limbs, as assessed through motion palpation, in both healthy and sick neonatal foals.

## 2 Materials and methods

### 2.1 Ethical approval

The study protocol was approved by CoBA - University of Bologna Animal Care and Use Committee (Approval number, 215,922; Approval date, 31 July 2023).

### 2.2 Animals' selection and population

This study included 28 foals born or hospitalized within the first days of life at the “*Stefano Belluzzi*” Perinatology Unit of the Department of Veterinary Medical Sciences of the University of Bologna, Italy, during the 2023–2024 foaling season. The population was divided into two groups: Group A included 12 healthy foals (11 Standardbred and 1 Westfalen) born from healthy mares with normal pregnancy, spontaneous and eutocic delivery, with Apgar score ≥8 (Austin, 2024) and a normal clinical evaluation during the entire study period. Group B included 16 sick foals (11 Standardbreds, 1 Italian Warmblood, 1 KWPN, 3 Quarter horses) suffering from flexural limb deformities (6/16), Perinatal Asphyxia Syndrome (PAS; 4/16), sepsis (4/16), prematurity/dismaturity (4/16), and dysphagia (1/16). Three out of 16 foals presented more than one diagnosis. Foals were defined as reported by Castagnetti et al. (2010). Regarding the type of medical assistance to foals, the level of care needed, from 1 to 3 (Level 1: minimum assistance; Level 2: additional assisted feeding; Level 3: intensive care), was recorded (Koterba, 1990). For each foal in Group B, the ability to stand or recumbency during the chiropractic evaluation was recorded. A written informed consent was given to the owner of each foal.

### 2.3 Chiropractic evaluation

**Fig. 1:**
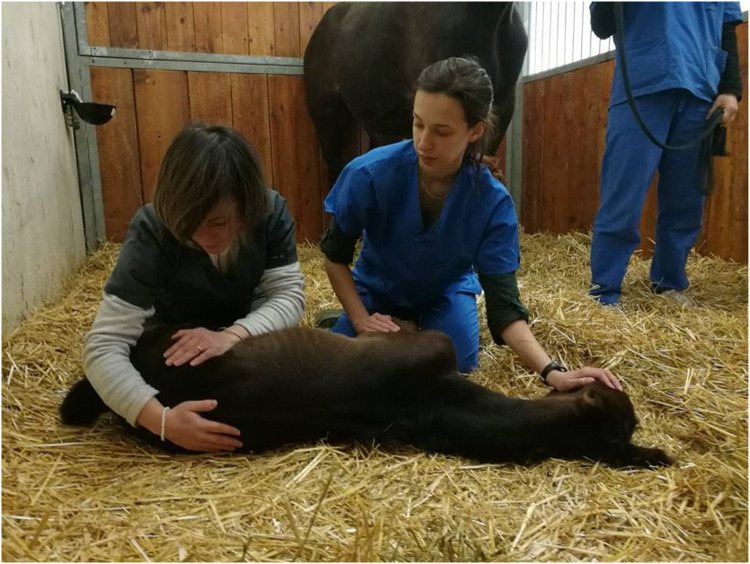
Motion palpation of the pelvis in a recumbent restrained foal.

Every foal was examined by an IVCA (International Veterinary Chiropractic Association) certified veterinarian (IA) assisted by a veterinarian student (SS) who gently restrained the foal, and an operator in charge of restraining the mare with halter and lead rope next to the foal (Fig. 1). The certified chiropractic veterinarian, not being a member of the Unit staff, was unaware of the animal's clinical condition, except obviously what was visually evident (i.e., intravenous catheter). The first chiropractic exam was performed between 1 and 10 days of age (mean 2.5 days). The exam was performed in a recumbent or standing position according to the foals’ clinical condition and ability to stand. During the chiropractic session, the foal was motion palpated by the application of a manual pressure and the assessment of the passive range of motion to locate the hypomobile motion units. The chiropractic evaluation was not painful for the animals and almost all foals were sleeping during the entire procedure that lasted 15 min for each side.

All the joints in the axial and in the appendicular skeleton were examined. The skeleton was divided into the following regions: cervical, thoracic, lumbar, sacroiliac, sacrococcygeal and lumbosacral, front limbs and hind limbs.

The hypomobile units were reported as chiropractic listings according to the Gonstead system (Cooperstein, 2003). The system designates the spatial orientation and motion of one articular segment in relation to adjacent segments (Eschbach et al., 2014). In details, according to the Gonstead system, a listed vertebral segment always referred to the motion unit consisting of that named segment and the cranial one, with the exception of the occiput, the atlas and the lumbosacral motion unit (called “intertransverse joint”). Despite the number of possible directions of movement for a motion unit (such as flexion, extension, axial rotation, right lateral flexion, left lateral flexion), the chiropractic exam defined only the dorsoventral flexion-extension and lateral flexion range of motion, since the axial rotation was coupled with the lateral flexion motion except for the occiput, the atlas and the lumbosacral motion unit (see Supplementary Material) (Cooperstein, 2003; Eschbach et al., 2014).

### 2.4 Statistical analysis

The odds ratio value was calculated to compare the frequency of the chiropractic alterations among the population of healthy and sick foals. The analysis included all the spinal and limbs joints. Only types of alterations observed at least in one subject (healthy or sick) were included in the calculation. Therefore, the total number of alterations, as well as the number of alterations calculated in a specific region does not represent the complete set of possible chiropractic alterations in the skeleton or in any given region. Confidence intervals (95 %) and P-values (*p* < 0.05) were generated.

## 3 Results

Clinical data such as breed, mare’s parity, sex, foal’s weight, Apgar score, diagnosis and level of intensive care, foal’s age at first treatment and foal’s position during treatment were described in Table 1. The chiropractic assessment of Group A revealed the presence of a total number of 112 joint motion alterations. In Group B, the total number was 305 (Table 2, Table 3).

**Table 1:** Clinical description of Group A and Group B.

Group	N.	Breed	Sex	Foal’s weight (Kg)	Mare’s parity	Apgar Score	Diagnosis	Level of intensive care	Foal’s age at first treatment (days)	Foal’s position during treatment
A	1	Standardbred	F	46	1	9	Healthy	–	10	Standing
A	2	Standardbred	M	58	3	9	Healthy	–	0	Standing
A	3	Standardbred	M	55	2	9	Healthy	–	3	Standing
A	4	Standardbred	F	50	4	10	Healthy	–	0	Standing
A	5	Standardbred	F	48	9	9	Healthy	–	0	Standing
A	6	Standardbred	F	54	2	9	Healthy	–	3	Standing
A	7	Westfalen	F	48	3	9	Healthy	–	0	Standing
A	8	Standardbred	F	45	8	9	Healthy	–	0	Standing
A	9	Standardbred	F	54	1	9	Healthy	–	4	Standing
A	10	Standardbred	F	59	3	10	Healthy	–	2	Standing
A	11	Standardbred	F	51	3	9	Healthy	–	3	Standing
A	12	Standardbred	F	54	3	9	Healthy	–	1	Standing
B	1	Standardbred	M	40	3	–	Flexural deformity	2	1	Recumbent
B	2	Quarter Horse	M	51	1	–	Sepsis (arthritis)	1	16	Standing
B	3	Standardbred	F	40	7	–	Prematurity Flexural deformity	3	10	Standing
B	4	Standardbred	M	45	1	–	Sepsis Flexural deformity	3	2	Recumbent
B	5	Standardbred	M	45	4	–	Flexural deformity	2	0	Recumbent
B	6	Standardbred	F	50	2	9	Dysphagia	2	0	Standing
B	7	Standardbred	F	53	5	10	Flexural deformity	1	0	Standing
B	8	Standardbred	M	55	9	–	PAS	3	3	Standing
B	9	Standardbred	F	42	1	6	PAS	2	0	Standing
B	10	Standardbred	M	42	4	5	PAS	3	2	Recumbent
B	11	Italian Warmblood	M	37	1	6	Prematurity PAS	3	2	Recumbent
B	12	Quarter Horse	F	23	2	10	Prematurity	2	0	Standing
B	13	KWPN	F	40	4	9	Dismaturity	2	0	Standing
B	14	Quarter Horse	M	38	1	–	Flexural deformity	3	2	Standing
B	15	Standardbred	M	60	3	–	Sepsis	3	3	Recumbent
B	16	Standardbred	M	53	4	8	Sepsis	1	5	Standing

**Table 2:** Number and percentage of alterations in Group A foals (*n* = 12).

Region	Number and percentage of alterations found in Group A	Number of foals with alterations
Cervical	32/192 (17 %)	
C4 body left		4/12
Occiput sup. Left/C6 body		2/12
Thoracic	23/492 (5 %)	
T3 Posterior		5/12
T6/T15 Posterior		0/12
Lumbar	16/180 (9 %)	
L2/L3/L6		0/12
L4		4/12
L5		3/12
Sacroiliac	29/132 (22 %)	
Sacral Base Post		5/12
Right PI Ilium		6/12
Front limbs	5/132 (4 %)	
Scapula, humeral head		1/12
Hind limbs	7/168 (4 %)	
Greater trochanter		3/12
Calcaneus		0/12

**Table 3:** Number and percentage of alterations in Group B foals (*n* = 16).

Region	Number and percentage of alterations found in Group B	Number of foals with alterations
Cervical	71/256 (28 %)	
C6 body left and C2		6/16
Occiput sup. left		9/16
C4 body left		9/16
Thoracic	91/656 (14 %)	
T4/T5/T8/T9		3/16
T10 posterior		4/16
T6 Posterior/T15 posterior		5/16
Lumbar	31/240 (15 %)	
L2/L5		4/16
L3		2/16
L4		1/16
L6		6/16
Sacroiliac	52/176 (30 %)	
Sacral Base Post		6/16
Right PI Ilium		7/16
Front limbs	22/176 (13 %)	
Scapula and humeral head		5/16
Hind limbs	32/224 (14 %)	
Greater trochanter		5/16
Calcaneus		5/16

Group B foals presented a significantly higher number of chiropractic alterations in the cervical, thoracic, lumbar and sacral regions and in both front and hind limbs compared to the Group A foals (Table 4).

**Table 4:** Results of the calculated Odds Ratio between Group A and Group B for each region.

**Region**	**Odds Ratio**	**Confidence interval 95%**	**P-value**
Cervical	1.9	1.2–3.1	0.0059
Occipital	4.4	1.2–15.8	0.0181
Occiput superior left	6.4	1.0–39.3	0.0338
C4 body left	2.6	0.5–12.2	0.2289
C6 body left	6.4	1.0–39.3	0.0338
Thoracic	3.3	2.0–5.3	<0.0001
T15	10.4	1.3–84.9	0.0090
Lumbar	1.9	1.0–3.5	0.0462
L6	5.0	1.0–25.5	0.0386
Sacral apex left	11.0	1.1–106.4	0.0195
Front limbs	3.6	1.3–9.9	0.0075
Hind limbs	3.8	1.6–8.9	0.0009

The predominant cervical alteration in both groups was C4 body left (hypomobility in right lateral flexion of the C3-C4 joint). Occiput superior left (hypomobility of the occiput in caudal right direction) and C6 body left (hypomobility in right lateral flexion of the C5-C6 joint) were the typical cervical alterations of sick foals.

The predominant thoracic alteration in Group A was T3 Posterior (hypomobility in extension or ventral direction of T2-T3 joint), while in Group B were T6 Posterior and T15 Posterior (hypomobility in extension or ventral direction of respectively the T5-T6 joint and T14-T15 joint). The lumbar area presented few alterations in both groups. In Group A, L2 and L3 did not show alterations, unlike L4 and L5. In Group B, the most represented alteration was L6 posterior and in 4 foals there were alterations in L2 posterior and L5 posterior.

Sacroiliac and sacrococcygeal alterations were distributed in both groups. In fact, Sacral Base Posterior (hypomobility of the sacrum in extension or ventral direction) and Right PI Ilium (hypomobility of the right tuber sacrale in ventro-cranial direction, decreased motion in extension of the sacroiliac joint) were frequently reported.

Front limbs presented only few alterations in Group A, while in Group B, 22 alterations regarding scapula and humeral head hypomobility were the most represented alterations.

Hind limbs were not highly involved. The only alteration in Group A was at the greater trochanter. In Group B, the alterations were more common: the greater trochanter and the calcaneus.

In Group B, sick recumbent foals were 6/16 and all of them showed at least an alteration in each examined region (Table 5).

**Table 5:** Number of alterations in Group B for each region, in recumbent and standing foals.

**Region**	**Number of alterations in recumbent foals (*n*=6)**	**Number of alterations in standing foals (*n*=10)**
Cervical	29	42
Thoracic	51	40
Lumbar	17	20
Sacroiliac	23	29
Front limbs	11	11
Hind limbs	17	15

## 4 Discussion

Since there is little research about chiropractic alterations in foals, the aim of this study was to evaluate the frequency and type of the chiropractic alterations assessed by motion palpation exam in healthy and sick neonatal foals.

In heathy foals, the most altered joints were in the cervical and sacroiliac regions. Cervical hypomobility could be a consequence of the positioning of the neck of the foetus during the last period of gestation, which is in a flexed position for most of the time (Ginther, 1998). Even after birth, the neck is probably stressed due to its length and to the position of the foal while suckling from the mare.

The sacroiliac region was the other most altered area, and this is very common in adult horses and even in humans, since it is subject to continuous mechanic stimuli (Tobolsky et al., 2016; Kurki, 2017; Persson-Sjodin et al., 2019; Toyohara et al., 2020). In horses, the kinetic energy comes from the propulsive action of the hind limbs, and it is distributed from the sacroiliac region to the spine. Moreover, newborn foals have an unstable posture and tend to maintain a wide based stance to cope with the poor motor control of the abductor and adductor muscles, shifting their weight in a mediolateral direction (Nauwelaerts et al., 2013). This kind of oscillations may lead to the sacroiliac alterations that have been found in this region. There is only one study about the prevalence of chiropractic alterations in healthy foals (Stroud et al., 2016). The authors evidenced the presence of a significant axial pelvic asymmetry from birth to 8–9 weeks of age in 10 healthy foals. The factors influencing the mobility of the pelvis in foals are still to be determined, but it can be hypothesized that, being already present at birth, hypomobility can be related to the birth process or the attempts to stand. In sick foals, therefore, a similar influence might be found.

The pelvic alterations in newborn foals may be related to the orientation and foetal movements in utero during the last months of pregnancy. According to Ginther’s research (Ginther, 1998), around the last month of pregnancy the fetal rear end is blocked into the pregnant uterine horn and the foetus can only flex and extend front and hind limbs, move the head and make movements of axial rotation with the cranial portion of its body. During delivery, only at the end of the expulsive phase, the hind limbs are extended and rotated with the pelvis in ventral position when the foal is almost out of the birth canal. The sacroiliac region is highly stressed by these movements being between the hind limbs compressed into the uterine horn and the cranial portion of the foal’s body, which rotates along the axis during delivery (Ginther, 1998).

The higher number of chiropractic alterations found in sick foals supports a possible relationship between health and the joint mobility. An important role may be played by aberrant somatovisceral reflexes can be found in sick foals due to visceral disfunction (Pikalov & Kharin, 1994). Stimuli coming from spinal and paraspinal structures can lead to a segmental reflex response of the nervous autonomic system, which can affect the visceral functions (Wisłowska, 1990; Haussler, 1999; Budgell, 2000). At the same time, the viscerosomatic reflexes should be considered: the visceral dysfunctions, mainly respiratory and digestive, can affect the somatic reflexes leading to muscles spasm and vertebral dysfunction (Pickar, 2002; Sonpeayung et al., 2018).

In sick foals, the most altered areas were the cervical, thoracic and sacroiliac joints. With regards to the cervical region, the same considerations for healthy foals would apply. Such alterations can be a result of the foetal positioning and the milking posture after birth, even though, not every foal was able to stand and milk from the udder.

It is hard to find an explanation to the relation between the Occiput Superior Left, C6 body left and atlas listing with the health status of the foals.

The thoracic vertebrae were widely altered in sick foals, both in recumbent and standing foals. One possible explanation of this finding can be found in the different biomechanics of respiration and pulmonary function in different body positions compared with healthy foals which probably spend more time in standing than sick ones (Katz et al., 2018). In humans, the volume of the rib cage is greater in the erect or sitting position compared to supine position where there is a greater diaphragmatic excursion and motion of the abdominal wall (Katz et al., 2018). Pulmonary function seems to improve with more erect posture in both healthy subjects and those with lung disease, heart disease, neuromuscular disease and obesity (Sonpeayung et al., 2018). It would be plausible to find similar differences involving the rib cage and the thoracic joints during the prolonged recumbency in sick foals. Furthermore, the different respiratory biomechanics in recumbent foals can affect the thoracic joints mobility.

A relationship between T15 and L6 with sickness is also difficult to justify. However, it is important to consider that the preganglionic fibers originating in the spinal cord connect to the celiac, cranial and caudal mesenteric ganglia; from these ganglia, the postganglionic fibers depart and innervate the abdominal viscera. The alterations may be correlated to a sympathetic dysfunction (Pikalov & Kharin, 1994).

While the sacroiliac listings did not highlight a specific relationship with healthy nor sick foals, the sacral apex hypomobility appeared related to sickness. The sacrum is connected with the coccygeal vertebrae and every sort of traction that it is made to restrain the foal for medical procedures and physical examinations can influence its mobility. Veterinarians and veterinary technicians usually wrap one arm around the foal’s neck from one side and with the other hand strongly pull its tail up from the base. The prevalence of sacral apex alterations in sick foals can find an explanation in the major number of exams and manoeuvres that these foals need during their hospitalization.

Other frequent alterations in sick foals were in the front limbs, especially scapula and humeral head regions, and to a lesser degree in the hind limbs, but it is important to underline that the most represented disease among sick foals was flexural limb deformity. This could be considered a limitation of the study, together with the different age at the time of examination, that could have influenced the chiropractic alterations found, except for the ones that may be caused by peripartum events (cervical and sacroiliac hypomobility) (Clayton & Townsend, 1989).

To overcome the limitations of the study, it would be important to include a larger number of animals and to divide them in subgroups based on age at the time of chiropractic evaluation and, most importantly, based on disease. This would make it possible to investigate the relationship between the different neonatal diseases and number and type of chiropractic alterations. Another approach could be to categorize the patients not by disease but considering the different dysfunctional organs and the presence of prolonged recumbency. It would also be important for chiropractic assessments to be conducted blindly. However, since the IVCA certified veterinarian was not a member of the Unit's veterinary staff, they were unaware of the animals' clinical classification.

## 5 Conclusion

The chiropractic assessment was useful to detect the difference in joint mobility between healthy and sick foals. In healthy foals, it may help to find the most stressed joints characterizing the neonatal period and address a suitable chiropractic treatment. A relationship between joint hypomobility and sickness was found, but the reasons cannot be determined given these preliminary results and the number of variables between groups.

This study lays the foundation for further investigations on the evolution of the hypomobility over time and after the adjustment, the difference in the affected areas by different neonatal disease and a possible role of chiropractic into the equine neonatal care.

In conclusion, these results can help developing a new neonatal integrated protocol including the chiropractic examination.

## Ethical statement

The study “Chiropractic evaluation in newborn foals: a preliminary study” was reviewed and approved by the Animal Care and Use Committee of Bologna University (Approval number, 215,922; Approval date, 31 July 2023).

## CRediT authorship contribution statement

**Ilaria Arena:** Writing – review & editing, Writing – original draft, Visualization, Methodology, Data curation, Conceptualization. **Silvia Signor:** Writing – review & editing, Writing – original draft, Visualization, Methodology, Formal analysis, Data curation. **Jole Mariella:** Writing – review & editing, Writing – original draft, Visualization, Software, Methodology, Formal analysis, Data curation. **Aliai Lanci:** Writing – review & editing, Writing – original draft, Visualization, Supervision, Data curation. **Francesca Freccero:** Writing – review & editing, Writing – original draft, Visualization, Supervision. **Castagnetti Castagnetti:** Writing – review & editing, Writing – original draft, Visualization, Supervision, Methodology, Data curation, Conceptualization.

## Declaration of competing interest

The authors declare that they have no known competing financial interests or personal relationships that could have appeared to influence the work reported in this paper.

## References

[bib0006] Alcantara J., Mayer D.M. (2008). The successful chiropractic care of pediatric patients with chronic constipation: A case series and selective review of the literature. Clinical Chiropractic.

[bib0005] Alcantara J., Anrig C.A., Plaugher G., Redwood D., Cleveland C.S. (2003). Fundamentals of chiropractic.

[bib0007] Alcantara J., Alcantara J.D., Alcantara J. (2015). The chiropractic care of infants with breastfeeding difficulties. Explore.

[bib0012] Alvarez C.B.G., L’ami J.J., Moffatt D., Back W., van Weeren P.R (2008). Effect of chiropractic manipulations on the kinematics of back and limbs in horses with clinically diagnosed back problems. Equine Veterinary Journal.

[bib0017] Austin S., Wong, Wilkins (2024). Equine clinical neonatology.

[bib0010] Borowitz S.M., Cox D.J., Tam A., Ritterband L.M., Sutphen J.L., Penberthy J.K. (2003). Precipitants of constipation during early childhood. The Journal of the American Board of Family Practice / American Board of Family Practice.

[bib0009] Browning M., Miller J. (2008). Comparison of the short-term effects of chiropractic spinal manipulation and occipito-sacral decompression in the treatment of infant colic: A single-blinded, randomised, comparison trial. Clinical Chiropractic.

[bib0030] Budgell B.S. (2000). Reflex effects of subluxation: The autonomic nervous system. Journal of Manipulative and Physiological Therapeutics.

[bib0018] Castagnetti C., Pirrone A., Mariella J., Mari G. (2010). Venous blood lactate evaluation in equine neonatal intensive care. Theriogenology.

[bib0035] Clayton H.M., Townsend H.G. (1989). Cervical spinal kinematics: A comparison between foals and adult horses. Equine Veterinary Journal.

[bib0003] Cleveland C.S., Redwood D., Cleveland C.S. (2003). Fundamentals of chiropractic.

[bib0008] Colloca C.J., Keller T.S. (2001). Electromyographic reflex responses to mechanical force, manually assisted spinal manipulative therapy. Spine.

[bib0020] Cooperstein R. (2003). Gontstead chiropractic technique (GCT). Journal of Chiropractic Medicine.

[bib0011] Driehuis F., Hoogeboom T.J., Nijhuis-van der Sanden M.W., de Bie R.A., Staal J.B. (2019). Spinal manual therapy in infants, children and adolescents: A systematic review and meta-analysis on treatment indication, technique and outcomes. PloS One.

[bib0021] Eschbach D., Spisak D., Bockhold H. (2014).

[bib0022] Ginther O.J. (1998). Equine pregnancy: Physical interactions between the uterus and conceptus. Proceedings of the American Association of Equine Practitioners.

[bib0002] Haldeman S. (2000). Neurologic effects of the adjustment. Journal of Manipulative and Physiological Therapeutics.

[bib0004] Haussler K.K. (1999). Chiropractic evaluation and management. The Veterinary Clinics of North America Equine Practice.

[bib0013] Haussler K.K. (2010). The role of manual therapies in equine pain management. The Veterinary Clinics of North America Equine practice.

[bib0015] Haussler K.K. (2016). Joint mobilization and manipulation for the equine athlete. The Veterinary Clinics of North America Equine Practice.

[bib0014] Haussler K.K. (2018). Equine manual therapies in sport horse practice. The Veterinary clinics of North America Equine practice.

[bib0034] Katz S., Arish N., Rokach A., Zaltzman Y., Marcus E.L. (2018). The effect of body position on pulmonary function: A systematic review. BMC Pulmonary Medicine.

[bib0019] Koterba A.M. (1990). Equine clinical neonatology.

[bib0024] Kurki H.K. (2017). Bilateral asymmetry in the Human pelvis. The Anatomical Record.

[bib0027] Nauwelaerts S., Malone S.R., Clayton H.M. (2013). Development of postural balance in foals. Veterinary journal (London, England : 1997).

[bib0025] Persson-Sjodin E., Hernlund E., Pfau T., Haubro Andersen P., Holm Forsström K., Rhodin M. (2019). Effect of meloxicam treatment on movement asymmetry in riding horses in training. PloS One.

[bib0033] Pickar J.G. (2002). Neurophysiological effects of spinal manipulation. The Spine Journal: Official Journal of the North American Spine Society.

[bib0029] Pikalov A.A., Kharin V.V. (1994). Use of spinal manipulative therapy in the treatment of duodenal ulcer: A pilot study. Journal of Manipulative and Physiological Therapeutics.

[bib0001] Sandoz R. (1976). Some physical mechanisms and effects of spinal adjustments. Ann Swiss Chiropractic Association.

[bib0016] Saunders D.G., Walker J.R., Levine D. (2005). Joint mobilization. The Veterinary clinics of North America Small animal practice.

[bib0032] Sonpeayung R., Tantisuwat A., Klinsophon T., Thaveeratitham P. (2018). Which body position is the best for chest wall motion in healthy adults? A meta-analysis. Respiratory Care.

[bib0028] Stroud R., Ellis J., Hunnisett A., Cunliffe C. (2016). A preliminary study to investigate the prevalence and progression of pelvic axial rotations among neonate foals. Journal of Veterinary Behavior: Clinical Applications and Research: Official Journal of: Australian Veterinary Behaviour Interest Group, International Working Dog Breeding Association.

[bib0023] Tobolsky V.A., Kurki H.K., Stock J.T. (2016). Patterns of directional asymmetry in the pelvis and pelvic canal. American Journal of Human Biology: The Official Journal of the Human Biology Council.

[bib0026] Toyohara R., Kurosawa D., Hammer N., Werner M., Honda K., Sekiguchi Y., Izumi S.I., Murakami E., Ozawa H., Ohashi T. (2020). Finite element analysis of load transition on sacroiliac joint during bipedal walking. Scientific Reports.

[bib0031] Wisłowska M. (1990). Back pain.

